# Dynamics of Macrophages and Polymorphonuclear Leukocytes Milk-Secreted by Buffaloes with Udders Characterized by Different Clinical Status

**DOI:** 10.3390/vetsci8100204

**Published:** 2021-09-22

**Authors:** Maria Chiara Alterisio, Paolo Ciaramella, Jacopo Guccione

**Affiliations:** Department of Veterinary Medicine and Animal Productions, University of Napoli Federico II, 80137 Napoli, Italy; mariachiara.alterisio@unina.it (M.C.A.); jacopo.guccione@unina.it (J.G.)

**Keywords:** water buffalo, mastitis, differential somatic cell count, host-response, innate immunity

## Abstract

The study evaluated the dynamics of macrophages and polymorphonuclear leukocytes milk-secreted by Mediterranean Buffaloes (MBs). Sixty quarter-milk-samples were collected and divided into three groups (*n* = 20 units each one): clinical mastitis (CM), subclinical mastitis (SCM), and intramammary infection (IMI). The control group consisted of an additional 20 healthy quarters. Their health status was assessed by clinical examination, quantitative somatic cell count (QSCC) and bacteriological milk culture. Finally, a differential somatic cell count (DSCC) was performed on all the milk samples. The mean percentage of macrophages, both in CM- and SCM-quarters, showed a significant difference as compared with the healthy-ones. Significant differences were also detected comparing the mean percentages of polymorphonuclear leukocytes between CM- and healthy-quarters, SCM and healthy, IMI and healthy. The QSCC revealed a weak-significant-negative-correlation with the quantitation of macrophages (r = −0.388), and a moderate-significant-positive-correlation with the polymorphonuclear leukocytes (r = 0.477). Macrophages and polymorphonuclear leukocytes showed a weak-significant-negative-correlation between them (r = −0.247). The interpretation of macrophages and polymorphonuclear leukocytes dynamics in milk provided beneficial information regarding the clinical status of the quarters enrolled. Future studies exploring the potential use of DSCC to improve udder health represent an interesting perspective in these ruminants.

## 1. Introduction

Mastitis can be considered one of the costliest and most prevalent diseases of the dairy Mediterranean buffaloes (MBs, *Bubalus Bubalis*). It has detrimental effects on animal health, welfare, and profitability of a dairy food chain able to produce an overall turnover of EUR ~1.2 billion per year, in Italy [1,2,3]. Although competencies on MBs’ mastitis are improving remarkably in the last years, buffalo medicine suffers from poor scientific knowledge compared to the bovine one [4]. In this context, the role of innate immunity in MBs affected by mastitis still represents one of the main unknowns [5,6]. As for other mammals, the latter constitutes the first line of host defence for the early recognition and removal of pathogens, as well as for the subsequent triggering of inflammation and adaptive response [7].

Two of the main milk-secreted cell populations, playing a relevant role within the mammary gland, are represented by macrophages and polymorphonuclear leukocytes (PMNL) [8,9]. Macrophages are phagocytic cells able to destroy bacteria through reactive oxygen species, nitric oxide, and lysosomal enzymes. They also remove bacterial and cellular debris, as well as milk contents, especially at the dry-off period [8,9,10]; finally, they promote the initiation of the inflammatory response by processing the bacteria, exposing the antigens on the membrane in association with type II histocompatibility molecules, and stimulating the rapid recruitment and the activity of the PMNL [8]. The latter instead defends the udder against the infecting bacteria mainly during the early stages of the inflammatory response [11]. Once the mammary gland is infected, an increase in the PMNL proportion is commonly seen; whether the host’s response results in complete bacterial elimination, a reduction in these cells amount can be detected within a few days, indicating the end of the acute stage [11].

Nowadays, the quantitative measurement of somatic cell count (QSCC) is an undisputed and well-established criterion to assess the host response and milk quality in MBs [2,5]. Although a value of SCC > 200 × 10^3^ cells/mL is currently accepted for diagnosis of mastitis [1,12,13,14], function and dynamics of immune cells in milk were never investigated in MB with different udder health statuses, assuming them to be similar to those of cows [15,16]. This condition magnifies the existing gap of knowledge between the two species [2,4,12]. Suffice it is to think that the differential somatic cell counts (DSCC) represent one of the most frequently used techniques to assess for udder health and to establish rational treatment strategies in dairy cows [17]. A deep host-response working knowledge against in-udder pathogens should represent a crucial requirement to improve udder’s health as well as to enhance the rational use of antibiotics and minimize the risk of antimicrobial resistance also in buffalo [2,4,12].

For all these reasons, the current investigation was initiated on dairy buffalo. We hypothesised that throughout the study of macrophages and PMNLs’ dynamic, in quarters characterized by different health statuses, a novel and in-depth clinical knowledge of the udder infections’ effects could be gained for the first time. Based on the previous considerations, the present study aims: (i) to compare the quantitative of macrophages and PMNL, obtained by the DSCC performed in quarters affected by mastitis with (clinical mastitis, CM) and without clinical signs (subclinical mastitis, SCM), intramammary infection (IMI) and in the healthy ones; (ii) to assess the correlations between values of QSCC, macrophages, and PMNL in order to define how the two leucocytes population change following the infections.

## 2. Materials and Methods

### 2.1. General and Ethical Animal Care

The current investigation was carried out during the springtime of 2020 (end of March-end of June 2021, to reduce possible seasonal influences) in a dairy farm located in Caserta district (southern Italy). It received institutional approval from the Ethical Animal Care and Use Committee of the University of Naples Federico II (n.PG/2017/0099607). Samplings were performed during the mastitis-monitoring program regularly practised by the farm. All the procedures performed during the study abide by the common good clinical practices [18]; moreover, the farmer was previously informed and in agreement about the purposes and methods of the present investigation. He also gave written consent for the study.

### 2.2. Farm and Study Population

The enrolled farm met the following eligibility criteria: (i) a housing and overall management system respecting the minimum welfare standard; (ii) animals belonging to late pregnant heifers and first lactation groups housed separately from the adults; (iii) a regular mastitis-monitoring program (e.g., including regular SCC monitoring, data analysis, etc.).

Therefore, an artificially induced seasonal calving herd (late winter–springtime), consisting of 850 MBs, was selected. It was characterized by herringbone parlour and buffaloes were milked twice a day. A 9-months average of 244 ± 25 (±standard deviation, SD) days in milk (DIM), milk yield of 2444 kg/head and bulk-milk somatic cell count values of 258 × 10^3^ ± 36 × 10^3^ cell/mL have been recorded at study time. As commonly observed in MBs’ farms, from late pregnancy to 40–50 DIM, all the first lactation MBs were housed separately from the adults and milked as first. After 50 DIM, these animals were progressively mixed with the adults, dropping the priority over milking time. Selected buffaloes were kept in a common paddock of ~875 m^2^ (~25 m × ~35 m). A common bedded area (with dried manure solids), a loafing area and a feeding alley with solid non-grooved concrete floor (cleaned once a day) characterized the barn. Milking animals were fed a total mixed ration including hay, silage, and a multi-vitamin integrator two times a day; free access to the protected water trough was always guaranteed.

For the purposes of the current study, *n* = 60 quarters milk samples belonging to first lactation MBs, ranged between 30 and 60 DIM, and positive in-udder specific bacteria have been collected. The sample size was calculated according to Friedman [19] considering a correlation analysis with 0.90 power level, a two-tailed significant level of 0.05, and an assumed effect size of 0.40. To promote homogenous comparisons between the different quarters’ health statuses, milk samples were clinically divided into three groups, made by *n* = 20 units each: affected by CM, by SCM or IMI. Finally, a control group of *n* = 20 healthy quarters was also chosen within the first lactation MBs group for the same purpose. The selection, according to the different quarters’ health status, has been performed by means of casual extraction within a study population made by all the quarters evaluated by the regular mastitis-monitoring program. All the quarters shared as common eligible criteria, including the absence of mastitis cases in a period between the late pregnancy and the sampling time.

### 2.3. Clinical Procedures and Udder Health Status

Immediately before samplings, all the buffaloes were individually submitted to a complete clinical examination with a particular focus on the teat and udder to assess clinical signs of disease [1,20]. All samples were aseptically collected according to the National Mastitis Council [21] guidelines, immediately after the discharge of 3-good-streams of milk, and during the regular evening milking. From each sampling, milk collected was divided into three aliquots: two (10 mL- BD Sterile Vacutainer, Oxford, UK) were used for the bacteriological milk culture (BMC, double culture to assign the correct bacteriological positivity) the QSCC; one was intended for flow cytometry (50 mL–BD Sterile Falcon Conical Tubes, Oxford, UK). Briefly, milk samples were placed in a cool box (4 °C) and brought to the reference laboratory within 1 h (h) of collection. As reported in our previous studies [2,14], the quarters’ health status has been assessed by a simultaneous evaluation of BMC, SCC, and clinical findings. Quarters having a positive BMC and producing milk with SCC > 200 × 10^3^ cells/mL have been classified as affected by mastitis (CM, with clinical signs–SCM without clinical signs). Those having positive BMC because of udder specific-pathogens (i.e., belonging to the contagious, environmental, or opportunistic categories) and milk with SCC < 200 × 10^3^ cells/mL have been defined as affected by IMI. As last, quarters with negative BMC and with SCC < 200 × 10^3^ cells/mL were classified as healthy.

### 2.4. QSCC and Bacteriological Investigations

The QSCC and BMC have been performed according to the diagnostic procedures used in our previous studies [1,2,12,14,20]. Briefly, samples have been promptly submitted to SCC analysis (Fossomatic 5000, Foss Electric, Hillerod, Denmark, approved for buffalo) and BMC within 2 h from the collection. The BMC and identification have been performed according to the National Mastitis Council guidelines [22]. Diagnostic investigations have been blindly performed by the laboratory (no information about the clinical statuses observed). Briefly, 10 μL of milk have been streaked on a blood agar plate (Merck KGaA, Darmstadt, Germany), incubated at 37 °C for 48 h and examined twice (at 24 and 48 h). Colonies are classified on a gross morphology basis. The number and type of colonies have also been assessed. As described in our previous studies [1,2,12,14,20], samples have been classified as contaminated when ≥3 dissimilar colonies have been identified. In order to differentiate between *Streptococci* and *Staphylococci, a* Gram staining and catalase test was carried out. The tube coagulase test has been instead used to classify coagulase-positive and coagulase-negative *Staphylococci.* Finally, a colourimetric system (Vitek 2 XL 120; bioMerieux Inc., Hazelwood, MO, USA) has been employed for the final identification of the microorganisms. The MacConkey agar (Oxoid, Basingstoke, UK) has been used for the *Enterobacteriaceae* grown; they were definitively classified by the same automatic system. Confidence levels greater than 0.90 have been used to identify the microorganisms at the species level; otherwise, they have been identified at the genus level.

### 2.5. DSCC

Isolation of macrophages and PMNL, antibody staining of milk cells, and flow cytometry analysis were performed according to Schwarz and collaborators [17], methodology. Briefly, the milk samples were centrifuged (15 min at 200× *g* and −10 °C) and cream layers and supernatants were discarded. Soon after, cell pellets were washed three times with 1% PBS and centrifuged. Non-conjugate monoclonal antibodies against CD11b and CD14 cell subsets, (VMRD Inc., Pullman, WA, USA), as well as APC and FITC labelled anti-IgG1 and IgG2b mouse IgG (Becton Dickinson PharMingen, San Jose, USA) were employed for identification of milk cells. Immune fluorescence and flow cytometry detection was performed by using a two laser-equipped *FACScalibur* apparatus (argon-ion and red-diode laser) and the *CellQuest* analysis software (Becton, Dickinson and co., medical equipment, New Jersey, USA) according to the guidelines reported by Schwarz and collaborators [17]. Briefly, five thousand cells from each sample were differentiated into macrophages, and PMNL. The PMNL were measured as CD11b+ cells, whereas macrophages as both CD11b+ and CD14+ cells. Outside the upper limit of background fluorescence were placed gates enclosing the antibody-positive cells. The negative control was represented by the cells with no antibody labelling and they were used as a measure for background fluorescence. In addition, isotype control antibodies were employed to assess the background staining (rat-IgG1, κ isotype control, 554686 and rat-IgG2a, κ isotype control, 554688; Becton, Dickinson and Co., Buena, NJ, USA).

### 2.6. Statistical Analysis

Data were expressed as absolute numbers, percentages, range or mean, ±SD, standard error (SE) and confidence interval (CI). Cell populations were analysed by standard descriptive statistics (QSCC and DSCC). Normality has been assessed by means of Shapiro Wilk tests, normal probability plots, and histograms. Log-transformation was applied to variables not normally distributed. Averages of macrophages and PMNL (above and below the threshold of 200 × 10^3^ cells/mL) were compared with a Two-Tailed Students’ *t*-test for independent variables. To avoid potential bias related to a fixed classification of quarter health status, the same test was employed to compare the overall proportion of quarters infected (i.e., including CM, SCM and IMI) with those healthy.

The QSCC, macrophages and PMNL observed at the different quarters’ health statuses have been instead compared with One-Way ANOVAs associated with a Tukey’s HSD test for post hoc multiple comparisons. Finally, the association between variables (i.e., overall QSCC, macrophages, and PMNL) has been assessed between *Pearson’s* correlations. The correlation coefficient’s values (*r*) of 0.00–0.10 have been considered as expression of a negligible correlation, while values of 0.10–0.39, 0.40–0.69, 0.70–0.89, 0.90–100 defined weak, moderate, strong and very strong correlations, respectively [23].

Probabilities < 0.05 for two-tailed results were considered statistically significant. All statistical data were analysed using dedicated software (SPSS, Version 26.0.0, Chicago, IL, USA).

## 3. Results

### 3.1. Clinical Procedures and Udder Health Status

The general examination of the udder did not reveal systemic signs of disease in any of the MBs affected by CM. Two MBs, each having one quarter affected by CM, lameness with the abduction of the limb adjacent to the painful quarter has been observed. The following clinical signs instead characterized the affected quarters (alone or concomitant): relative asymmetry due to enlargement, hardness, warmness and pain, teat hyperkeratosis scored from 1 (mild) to 5 (severe), watery milk and clots.

### 3.2. QSCC and Bacteriological Investigations

Overall, the *n* = 80 udder quarters selected showed a QSCC ranged between 4.01 and 7.39 Log10 cells/mL (average of Log10 cells/mL = 5.43, SE = 0.89, CI = 5.25–5.60). Dividing the QSCC by the cut-off of 200 × 10^3^ cells/mL, above the threshold they were ranging between 5.34 and 7.39 Log10 cells/mL (average of Log10 cells/mL = 6.08, SE = 0.87, CI = 5.90–6.26), while below they between 4.01 and 5.30 (average of Log10 cells/mL = 4.77, SE = 0.53, CI = 4.66–4.88). A statistically significant difference has been observed between the mean QSCC values above and below, the threshold considered (*p* < 0.0001). Analyzing the data according to the different quarters’ health status, those affected by CM showed values between 6.07 and 7.39 Log10 cells/mL, while those with SCM between 5.34 and 5.94 Log10 cells/mL. Considering the IMI and healthy status, they were instead characterized by a range of 4.16 and 5.30 Log10 cells/mL, and of 4.01 and 5.21 Log10 cells/mL, respectively. The mean values observed are reported in detail in Table 1. Concerning the BMC, a full correspondence between the double investigations has been detected. Overall, from 60 positive samples, 87 mastitogens-bacteria for MBs have been isolated. *Staphylococcus aureus* was the most frequently observed bacteria (28/87–32.2%), followed by *Staphylococcus haemolyticus* (13/87–14.9%), *Staphylococcus chromogenes* (11/87–12.6%), *Staphylococcus xylosus* (10/87–11.5%), *Aerococcus viridans* (9/87–10.3%), *Streptococco dysgalactie* (9/87–10.3%), and *Staphylococcus warneri* (7/87–8.0%). Details regarding mono- and co-infection states are reported in detail in Supplementary file–Table S1.

### 3.3. DSCC

**Scheme 1:**
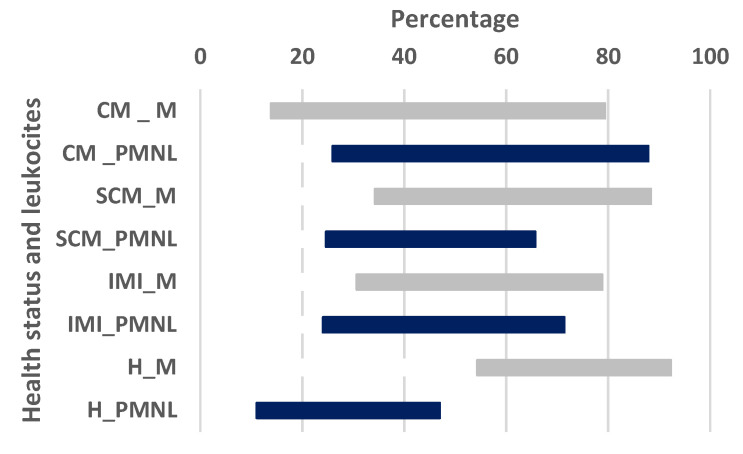
Gantt graph displaying the range values of macrophages and polymorphonuclear leukocytes milk-secreted by dairy buffalo with different udder health status. Legend: CM = clinical mastitis; SCM = subclinical mastitis; IMI = intramammary infection; H = healthy; M = macrophages; PMNL = macrophages and polymorphonuclear leukocytes.

Overall, the *n* = 80 udder quarters selected showed a range of macrophages between 13.87 and 92.30% (average % = 58.13, SE = 1.81, CI = 54.59–61.82). Dividing the macrophages by the cut-off of 200 × 10^3^ cells/mL, above the threshold, they ranged between 13.87 and 88.35% (average % = 52.75, SE = 2.69, CI = 44.80–55.70), while below they between 20.65 and 92.30% (average % = 63.51, SE = 2.08, CI = 59.30–67.73). A statistically significant difference has been observed between the two values (*p* < 0.01). Comparing the mean macrophages value observed in quarters positive for mastitogens bacteria (affected by IMI, SCM and CM–average % = 54.43, SE = 2.08, CI = 50.25–58.61) with that detected in healthy quarters (average % = 69.25, SE = 2.28, CI = 64.77–74.33) a statistical difference was also pointed out (*p* < 0.05). The results of the analysis according to the different quarter health status are also reported in Scheme 1. Briefly, those affected by CM showed values between 13.87 and 79.37%, while those with SCM between 34.25 and 88.35%. Considering the IMI and healthy status, they were instead characterized by a range of 30.65 and 78.81%, and of 54.29 and 92.30%, respectively. The mean values and comparisons are reported in detail in Table 1. Briefly, a significant difference for the mean results has been observed between CM quarters and healthy (*p* < 0.0001). Looking to the SCM quarters, they show a significant difference with healthy (*p* < 0.05).

Concerning the PMNL, the *n* = 80 udder quarters selected showed an overall range between 11.07 and 81.84% (average %, = 43.14, SE = 1.88, CI = 39.38–46.90). Dividing the PMNL by the cut-off of 200 × 10^3^ cells/mL, above the threshold they were ranged between 24.64 and 81.84% (average% = 50.25, SE= 2.69, CI = 44.80–55.70), while below they were between 11.07 and 71.37 (average % = 36.03, SE = 2.14, CI = 31.68–40.37). A statistically significant difference has been observed between the two values (*p* < 0.0001). Comparing the mean PMNL value observed in quarters positive for mastitogens bacteria (affected by IMI, SCM and CM–PMNL average % = 47.56, SE = 2.10, CI = 43.35–51.76) with that detected in healthy quarters (PMNL average % = 29.88, SE = 2.42, CI = 24.80–34.96) a statistical difference was also pointed out (*p* = 0.01). The results of the analysis according to the different quarter health status are also reported in Scheme 1. Briefly, those affected by CM showed values between 25.92 and 81.84%, while those with SCM between 24.64 and 65.67%. Considering the IMI and healthy status, they were instead characterized by a range of 24.04 and 71.37%, and of 11.07 and 46.98%, respectively. The mean values and comparisons are reported in detail in Table 1. Briefly, a significant difference for the mean results has been observed between CM quarters and healthy (*p* < 0.0001). Looking at the SCM quarters, they showed a significant difference with healthy (*p* < 0.01), while IMI revealed a difference with the healthy ones (*p* < 0.01).

**Figure 1:**
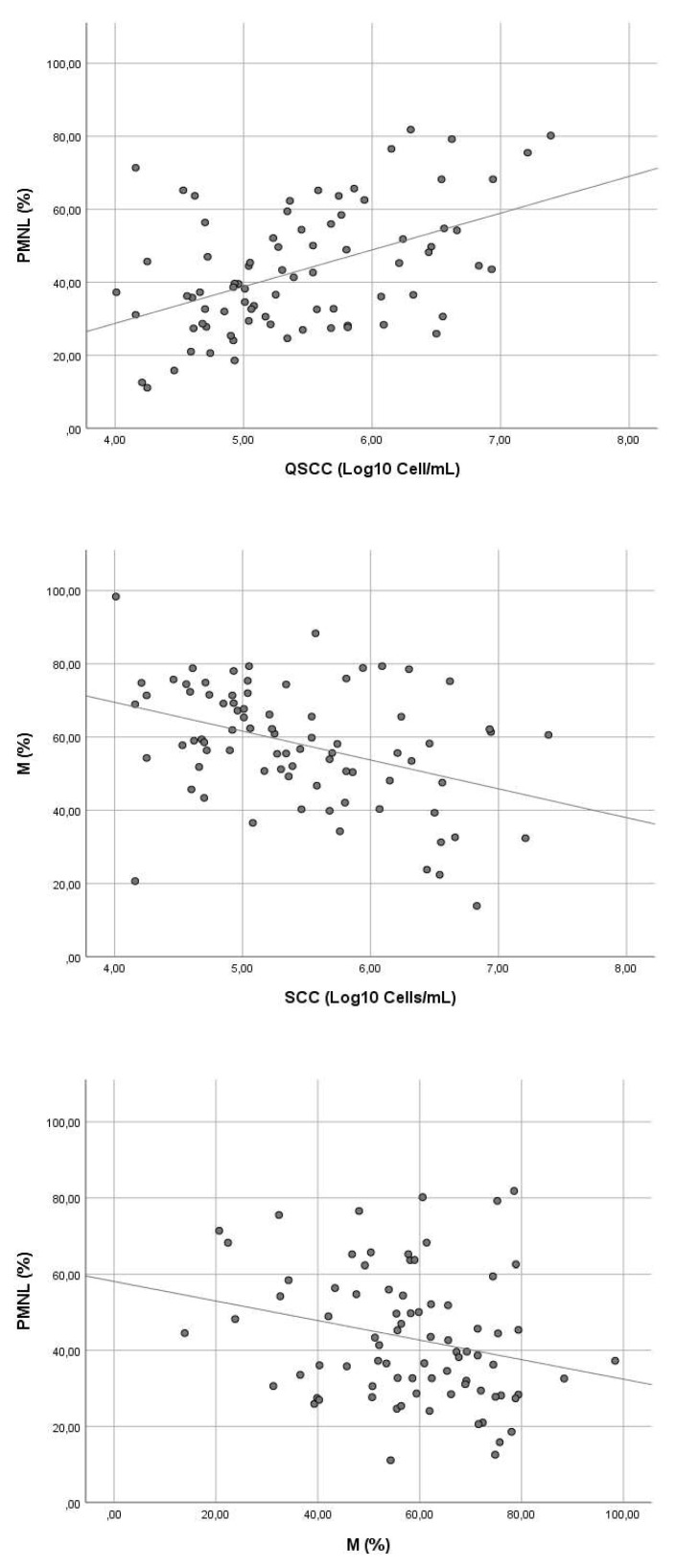
Scatter plots displaying the relationship between. different variables. PMNL vs. QSCC, r = 0.477–*p* < 0.0001; M vs. QSCC, r = −0.388–*p* < 0.0001; and M vs. PMNL r = −0.247–*p* = 0.027. Legend: PMNL = polymorphonucleates; % = percentage; QSCC = quantitative somatic cell count; Log = logarithm; mL = milliliters; M = macrophages.

Analyzing the correlation between the overall variables (Figure 1), the overall QSCC was weakly negative-correlated to the quantitative of macrophages (*r* = −0.388; *p* < 0.0001), while moderately positive to the amount of PMNL (*r* = 0.477; *p* < 0.0001). Moreover, macrophages and PMNL showed a weak negative correlation between them (*r* = −0.247; *p* = 0.027).

## 4. Discussion

Estimation under field conditions of the DSCC usefulness can represent a stimulating challenge in buffalo medicine, where the knowledge regarding the host-response in animals with different udder health statuses was never explored, so far.

The decision to enrol the first lactation MBs originating from the same herd, during a restricted period, arises from the necessity (i) to avoid potential influences on the host response due to chronic infections originating from previous lactations [1,12,13,20]; (ii) to evade the consistent metabolic and inflammatory changes observed in multiparous buffalo around calving, potentially able to influence function and dynamics of the milk’s cells [24,25]; (iii) to minimize the adverse effects driven by others key animal- (e.g., the number of lactations, DIM, sampling time, etc.) and herd-factors (e.g., distributions of IMI-causing pathogens, levels of IMI, genetic differences, etc.) with a role not fully elucidated even in dairy cows [26].

Across the study, the most frequently isolated causative agent (32.2% of times), with a high percentage of mono-infection cases (Supplementary file–Table S1) was the *S. aureus*. As observed in our previous studies, the microorganism has been often detected as the most representative Gram-positive in-udder pathogen [1,2,12,14]. It was related with high contagiousness, mono-infection mastitis and economic losses due to its negative influence on milk quality and yield in dairy MBs [1,2,12,14]. Considering the overall BMC results, the high-frequency isolation of *S. aureus* prevailed over the non-aureus staphylococci (NAS) (Supplementary file–Table S1). A similar impact has been described both in MBs [2,12,20] and cows [27], where the isolation of NAS and Streptococci has been considerably lower in herds simultaneously infected by *S. aureus* genotypes characterized by high contagious potential [28].

The data analyses allowed us to explore the existing differences between the two main milk-secreted cells (macrophages and PMNL) produced by quarters above (CM and SCM) and below (IMI and healthy) the quantitative threshold of normality, for the first time. In 2006, Moroni and collaborators [29] observed that quarters with SCC > 200 × 10^3^ cells/mL were always infected in their study population. Tripaldi and collaborators [15] improved the accuracy of the clinical deduction, concluding that SCC > 200 × 10^3^ cells/mL could be used for the early identification of buffaloes affected by mastitis [15]. After these first two examples, this quantitative threshold was used by most of the clinical investigations present in the literature [1,2,12,13,14,20]. Looking at the current data, they seem to confirm the presence of different host responses above and below the cut-off considered (*p* < 0.0001). A different proportion of leukocytes between healthy and infected animals has long been defined in dairy cows [30]. Indeed, the PMNLs commonly predominate in the presence of mastitis triggered by resident cells (lymphocytes, macrophages, and epithelial cells), while an uninfected mammary gland is commonly characterized by a high proportion of macrophages [9,17,31,32]. According to the results of our analysis, the negative correlation between macrophages and PMNLs enable to suppose a similar dynamic also in dairy MBs (r = −0.247, *p* = 0.027, Figure 1), since the cell population milk-secreted changes in favour of the PMNL as soon as the quarters become infected. Moreover, if the clinical status worsens and the QSCC increases, macrophages tend to decrease (r = −0.388, *p* < 0.0001) while a contrary trend is observed in PMNL (r = 0.477, *p* < 0.0001) (Figure 1). The finding seems to agree with what was recently observed by Damm and collaborators [9] in dairy cows, who demonstrate a decrease in the macrophages proportion (r = −0.3400, *p* < 0.001) and an opposite trend of the PMNL (r = 0.3604, *p* < 0.001) as QSCC increased. Unfortunately, due to the cross-sectional nature of the study, relevant information regarding cellular dynamics over time was not gained. As happened for dairy cows [33], further longitudinal research should be performed to address the short-coming, evaluating the changes of the different leukocytes throughout the stages of the disease (e.g., MBs moving from infection to acute/chronic mastitis and vice versa returning to healthy levels) and assessing the major risk factors affecting the values observed.

Despite the previous clinical deductions, it is necessary to underline how recent studies in cows revealed apparent inflammatory reaction based on an elevated proportion of PMNL at very low QSCC values [17,31,32]. Schwarz and collaborators [17] described this phenomenon for the first time in dairy cows in 2011, discovering a PMNL proportion up to 74.43% in six udder quarters characterized by a QSCC from 9000 to 46,000 cells/mL. Interestingly, an analogous observation was made in our study in two quarters (PMNL = 71.37% and 65.21%) having QSCC widely below the threshold of normality (21,011 and 33,665 cell/mL, respectively). As proved in previous studies of the dairy cow, the constant pressure from in-udder microorganisms and/or the initial phase of inflammatory status may have triggered the elevated proportion of PMNL, even in the presence of low QSCC values [9]. The outcome suggests that a clear host response (indicative of active infection) can appear with a QSCC clearly below the cut-off value of 200,000 cells/mL also in MB. The finding receives further confirmation from the results of the comparisons between positive quarters to specific in-udder pathogens (i.e., affected by IMI, SCM and CM) and healthy ones. Even in this case, the differences observed may be justified by the presence of an active host response that starts from the status of IMI characterized by a mild severity of the process [9]. These outcomes might question the usefulness of the quantitative cut-off of 200,000 cells/mL in MBs. Although the latter has been considered as reliable so far, the observation of the PMNL as predominant cell population in quarters affected by mild IMI suggests that a lowering of the limit may improve QSCC’s diagnostic sensitivity, as observed for dairy cows [17]. The hypothesis requires further studies both to confirm the findings and to define the dynamic of the two cell populations within smaller cellular ranges to set out the presence of recurrent patterns and potentially to establish a new limit for the MBs.

Finally, a further interesting point of discussion is represented by the proportions of the cells within the study population (Scheme 1, Table S1). The overall percentage of PMNL (range = 11.07–81.84%, average % = 43.14, SE = 1.88, CI = 39.38–46.90) can be considered similar to that recently described for dairy cows (range = 13.00–92.00%, mean ± SD = 58.68 ± 16.19) [9], while that of macrophages (range = 13.87–92.30%, average% = 58.13, SE = 1.81, CI = 54.59–61.82) have been found as slightly higher (range = 7.00–79.00%, mean ± SD = 35.45 ±14.30) [9]. A subtle difference of this cell component may also be hypothesized since the proportion of the macrophages secreted from healthy quarters in MBs (average% = 69.25, SE = 2.28, CI = 64.77–74.33) consisted of higher values as compared to that observed in cows with similar health status (23.06%) [17]. As mentioned before, the ability of macrophages to assist local inflammatory processes, attracting the PMNL and inducing the bactericidal activities of neutrophils, is believed to be of great importance as a defence mechanism [26,33]. In dairy cows, the severity and lifespan of new infections are influenced by the promptness of the leucocytes migratory response facilitate by the macrophage’s efficiency [26,33]. A more responsive host-response, supported by a greater quantity of macrophages, may help to explain why IMI and SCM seem less prone to progress to CM in MBs as compared to the cow [1,2,5,14]. Although this interesting hypothesis may suggest a difference in the innate immunity of the two dairy species, it needs further explorations to be confirmed.

On the one hand, our study opens new exciting clinical considerations regarding two of the most representative milk-secreted cells population. On the other hand, it presents some limitations. The first is represented by the lack of data regarding the lymphocytes, a cell population necessary for the initiation and suppression of the immune response [9], while the second is related to a limited number of samples enrolled. Although it could be considered as a first investigation on the role and dynamic of two of the key elements of the intramammary immune response, a deeper knowledge of the host response requires further studies, including the remaining cells not explored yet and a bigger study population.

## 5. Conclusions

The current clinical study allows promising clinical-diagnostic considerations regarding the use of DSCC in MBs, for the first time. The flow cytometry seems to provide accurate information about the real state of the buffalo quarters, revealing evidence for inflammatory reactions just above the 20,000 cells/L. On the one hand, the relationship between the two cell populations studied (macrophages and PMNL) and the QSCC appears comparable to what was observed in dairy cows. On the other hand, the higher proportion of macrophages detected in the healthy quarters represents one of the main differences detected. As described for dairy cattle and also in MBs, new and rational udder health and milk quality monitoring programs can be produced using verified studies such as this one, to gain complete knowledge of the role and dynamics of the host response to infections. Although our study allows new and exciting clinical considerations regarding two of the most representative milk-secreted cell populations, more complete studies should be performed to improve scientific knowledge focused on dairy Mediterranean buffalo.

## Figures and Tables

**Table 1:** Quantitative and differential somatic cell counts in first lactation dairy buffaloes with different quarters health status expressed as average results.

**QSCC (Log10 Cells/mL)**						
	*Ave.*		*S.E.*		*C.I.*	
**CM**	6.55		0.08		6.38–6.71	
**SCM**	5.62		0.04		5.53–5.70	
**IMI**	4.88		0.06		4.74–5.02	
**H**	4.67		0.07		4.50–4.83	
**DSCC (%)**						
	**Macrophages**			**Polymorphonuclear**		
	*Ave*.	*S.E.*	*C.I.*	*Ave*.	*S.E.*	*C.I.*
**CM**	49.08 ^a^	4.28	40.11–58.05	53.97 ^a^	4.11	45.36–62.57
**SCM**	56.42 ^c^	3.16	49.80–63.04	46.54 ^e^	3.37	39.46–53.61
**IMI**	57.79	3.15	51.18–64.39	42.17 ^e^	3.01	35.86–48.48
**H**	69.25 ^b,d^	2.28	64.77–74.33	29.88 ^b,f^	2.42	24.80–34.96

Legend: QSCC = quantitative somatic cell count; Log = logarithm; mL = millilitres; ave = average; S.E. = standard error; C.I. = confidence interval CM = clinical mastitis; SCM = subclinical mastitis; IMI = intramammary infection; H = healthy; DSCC = differential somatic cell count; % = percentage; ^a,b^ *p* < 0.0001; ^c,d^ *p* < 0.05, ^e,f^ *p* < 0.01. The statistical differences regarding percentages of macrophages and polymorphonuclear cells have to be considered within the columns.

## Data Availability

All the data supporting reported results can be found into the manuscript.

## Supplementary Materials

The following are available online at https://www.mdpi.com/article/10.3390/vetsci8100204/s1.

## Abbreviations

BMC	Bacteriological milk culture
CI	Confidence Interval
CM	Clinical mastitis
DIM	Days in milk
DSCC	Differential somatic cell count
IMI	Intramammary Infection
MBs	Mediterranean Buffaloes
NAS	Non-aureus Staphylococci
PMNL	Polymorphonuclear leukocytes
QSCC	Quantitative somatic cell count
SCM	Subclinical Mastitis
SE	Standard Error
SD	Standard Deviation
CI	Confidence Interval
