# Supplementary material for: Dynamics of Macrophages and Polymorphonuclear Leukocytes Milk-Secreted by Buffaloes with Udders Characterized by Different Clinical Status
Source: Vet Sci. 2021 Sep 22;8(10):204. doi: 10.3390/vetsci8100204 (PMC8539079; doi:10.3390/vetsci8100204)
Supplement: Supplementary file 1 [file vetsci-08-00204-s001.zip › vetsci-1307933-SI.pdf]

Table S1. Number and percentage of mastitogens bacteria isolated in mono- and co-infection from milk samples belonging to the study population.

Mono-infection	***CM***		***SCM***		***IMI***	
	n	*%*	*n*	*%*	*n*	*%*
S. aureus	4	6.7	9	15.0	7	11.7
S. haemolyticus	3	5.0	1	1.7	-	-
S. chromogenes	-	-	-	-	-	-
S. xylosus	3	5.0	3	5.0	-	-
A. viridans	-	-	-	-	-	-
S. warneri	-	-	-	-	-	-
Str. Dysgalactie	3	5.0	-	-	-	-

Co-infection		***CM***		***SCM***		***IMI***	
		n	*%*	*n*	*%*	*n*	*%*
S. aureus	*A. viridans*	-	-	2	3.3	2	3.3
S. aureus	*S.haemolyticus*	-	-	1	1.7	-	-
S. aureus	*S. xylosus*	-	-	-	-	2	3.3
S. aureus	*S.chromogenes*	-	-	-	-	1	1.7
S. aureus	*S.haemolyticus*	-	-	-	-	3	5.0
S.haemolyticus	*A. viridans*	2	3.3	-	-	-	-
S.haemolyticus	*S.chromogenes*	1	1.7	-	-	-	-
S.haemolyticus	*S. warneri*	-	-	-	-	2	3.3
S.chromogenes	*Str.dysgalactie*	2	3.3	-	-	-	-
S.chromogenes	*S. warneri*	2	3.3	1	1.7	-	-
S.chromogenes	*S. xylosus*	-	-	1	1.7	-	-
S. xylosus	*A. viridans*	-	-	-	-	3	5.0
S. xylosus	*S. warneri*	-	-	1	1.7	-	-
Str.Dysgalactie	*S. warneri*	-	-	1	1.7	-	-

Legend: *CM* = clinical mastitis; *SCM* = subclinical mastitis; *IMI* = intramammary infection; n = number; *%* = percentage; «-» = *not detected; S. aureus = Staphylococcus aureus; A. viridans = Aerococcus viridans; S. haemolyticus = Staphylococcus haemolyticus; S. xylosus = Staphylococcus xylosus; S. chromogenes = Staphylococcus chromogenes; S. warneri = Staphylococcus warneri; Str. Dysgalactie = Streptococcus dysgalatie.*
